# Reduced polyethylene wear in dual mobility versus single mobility hip implants: Results from quantitative and qualitative scanning electron microscopy analysis

**DOI:** 10.1002/jeo2.70177

**Published:** 2025-02-26

**Authors:** Antonio Pompilio Gigante, Marco Senarighi, Carlo Ciccullo, Luca De Berardinis, Luca Farinelli

**Affiliations:** ^1^ Clinical Orthopedics, Department of Clinical and Molecular Sciences, School of Medicine Università Politecnica delle Marche Ancona AN Italy; ^2^ IRCCS INRCA Ancona AN Italy

**Keywords:** dual mobility cup, polyethylene mobile component, total hip arthroplasty, ultra‐high molecular weight polyethylene, wear

## Abstract

**Purpose:**

Dual mobility cup in total hip arthroplasties has gained popularity worldwide as it reduces instability providing greater range of motion. However, increased polyethylene wear remains debated. This in vitro study aimed to measure and qualitatively analyse the wear of ultra‐high molecular weight polyethylene in contemporary dual mobility cup compared to conventional single mobility acetabular component.

**Methods:**

Hip simulator was used to compare ultra‐high molecular weight polyethylene wear in dual mobility and single mobility acetabular component The specimens were tested at an in vitro angle of 30° relative to the ISO standard fixated position. Flexion/extension, abduction/adduction, and internal/external rotation were simulated. Testing was stopped every 0.5 million cycles until 5.0 million cycles were reached and the liners were disassembled and weighted to assess mass loss. The test fluid was sent for scanning electron microscopy analysis and wear particles were characterized for mean equivalent circle diameter, form factor, maximum Feret diameter, minimum Feret diameter, area, perimeter and aspect ratio.

**Results:**

Dual mobility hip reported a lower wear respect to single mobility (20.4 and 39.6 mg/Mcy, *p* < 0.01). Moreover, conventional acetabular component produced wear particles with higher equivalent circle diameter, area, perimeter, minimum and maximum Feret diameter, while aspect ratio and form factor resulted higher in dual mobility polyethylene wear. No cases of ultra‐high molecular weight polyethylene rupture have been reported.

**Conclusion:**

Dual mobility cup produces less wear than conventional single mobility acetabular component ensuring lower risk of instability and greater range of motion. Further studies are needed to definitively clarify the issue of wear in the dual mobility prosthesis.

**Abbreviations**
A: area
AR: aspect ratio
DM: dual mobility
ECD: mean equivalent circle diameter
EDTA: ethylenediaminetetraacetic
FF: form factor
ISO: International Organization for Standardization
n.s.: not significant
P: perimeter
PE: polyethylene
SD: standard deviation
SEM: scanning electron microscopy
SM: single mobility
THA: total hip arthroplasty
UHMWPE: ultra‐high molecular weight polyethylene
XLPE: highly cross‐linked polyethylene

## INTRODUCTION

Total hip arthroplasty (THA) is considered as one of the most successful surgical procedures in orthopedics, capable of relieving pain and improving the quality of life in patients with end‐stage hip arthritis who do not respond to other treatments [4, 11, 16, 26, 27]. As healthcare continues to improve and life expectancy increases, the demand for total and partial joint replacements will grow as the world's population grows older and more active. The number of primary THA's performed in United States in 2022 was nearly 952,000 [18].

Over the years, various mechanisms have been developed to reduce surgical invasiveness and mechanical complications such as post‐operative instability of THA. The incidence of instability after THA in the primary and revision setting has been reported as high as 7% and 25%, respectively [27]. The risk factors for instability after THA are multifactorial and can be divided into patient‐specific (gender, age, adductor deficit) or related to operative variables (surgical approach, malposition of the component, femoral head diameter) [19, 29].

Approximately 50% of dislocations occur within the first 3 months after the procedure, and more than 75% occur within the first year [6].

Dual‐mobility (DM) acetabular component prostheses have recently gained increased attention worldwide as an alternative option in the prevention of instability in primary and revision THA. They offer the advantage of increased stability without compromising clinical results even though the literature has not yet provided definite and widespread data on implant longevity [25, 28, 33].

In France, the country where the DM cup was developed, the dislocation rate has globally decreased from 9% in 2005 to 6% in 2014 [7].

The double‐articulation cup was created by Professor Gilles Bousquet and André Rambert (engineer) in 1974. The original idea combined the ‘low friction’ principle of THA popularized by Charnley with the McKee‐Farrar concept of using a larger diameter femoral head to improve implant stability [8, 24].

The first design (Novae‐1®) showed a metal head with a diameter of approximately 22.2 mm that articulated with a polyethylene (PE) liner, which in turn articulated with an acetabular shell. This shell was composed of stainless steel and coated with Al_2_O_3_ in a cylindrical/spherical configuration.

First‐generation DM cups relied upon press‐fit fixation consisting of two pegs that were driven into the pubis and ischium, as well as a screw in the dome.

Over time, the development of new technologies has led to changes in the design of the double mobility. The later generations were in fact not coated with Alumina, but a double layer of hydroxyapatite and plasma titanium to provide a more three‐dimensional surface for osteointegration.

Other modifications involved the shape of the cup, making it less prominent anteriorly, to improve fixation and prevent irritation of the iliopsoas tendon, and the change in the acetabular mobile bearing, introducing the ultra‐high molecular weight PE (UHMWPE) and then the highly cross‐linked PE (XLPE) to reduce linear and volumetric wear [13, 32].

Linear and volumetric wear in PE are critical parameters for assessing the longevity and performance of hip implants, as they directly influence the generation of wear particles, the risk of osteolysis, and the overall lifespan of the prosthetic components.

The DM prosthesis consists of two distinct joints: a small joint between the femoral head and the PE bearing and a large joint between the PE head and the acetabular shell. Most of the movement occurs in the small joint. Movement in the large joint occurs only when the neck of the stem meets the PE head. Wear can occur at three interfaces: the small and large bearing and the neck‐PE contact area (referred as the ‘third joint’) [23, 31].

The range of motion without impingement within the DM cup is increased due to the third articulation compared to a conventional THA. This articulation in fact engages the motion of the mobile PE component at the large joint at the time of femoral neck contact with the bevel.

Previous biomechanical studies have shown that motion and wear within the DM implant predominate in the small joint, but these studies were not comparative and only evaluated the potential wear performance on THA explants with suboptimal in vivo function and/or PE damage to the movable component that could occur at the time of revision [1, 9, 13, 32]

There is a need to better understand, quantify and predict DM cup wear. We recreated a hip model in the laboratory to simulate conventional motion and biomechanical function of the hip in the laboratory.

To the best of our knowledge, this is the first study that aims to evaluate the quantitative and qualitative difference in PE wear between DM and conventional single‐mobility (SM) cups, using a scanning electron microscopy (SEM) analysis.

## MATERIALS AND METHODS

### Wear test

A servo hydraulic six station hip simulator (Endolab®) was used for wear test.

All tests were performed according to the normative references ISO 14242‐1:2014/Amd 1:2018, ISO 14242‐2:2016 using the parameters specified in Table 1 [20, 21].

**Table 1:** Test parameters.

Parameter	ISO 14242‐1
Force curve	Double‐peak according to PAUL
Load direction is fixed relatively to	Insert
Force maximum	3.0 kN
Frequency	1.0 Hz
Insert inclination ‘L’ according to ISO 14242‐1 (reference position)	30°^a^
Inferior inclination of the insert axis to the head axis (reference position)	0°
Flexion/etension	+25°/−18°
Abduction/adduction	−4°/ + 7°
External/internal rotation	−10°/ + 2°
Test fluid	Calf serum
Test fluid temperature	37°C + /− 2°C
Number of cycles	5.0 milion^b^

^a^ Corresponds to an in vivo inclination of 45°.

^b^ Test fluid replaced every 0.5 million cycles.

The DM cup NOVAE E 69 TH (Serf) and the conventional single mobility cup HYPE 51 C (Serf) were tested for wear production with an UHMWPE: CI 69/28 E and HIPER 28 C (Serf), respectively. Ten specimens were tested in each group with a 28 mm diameter Stainless Steel (ISO 5232‐9) femoral head.

The acetabular components (metal back/insert) were tested at an in vivo angle/inclination of 45° in the coronal plane (angle between the front face of the acetabular component and the horizontal), which corresponds to an angle *L* = 30° relative to the ISO standard fixated position used for in vitro testing. The resulting hip joint force was applied via the metal back shell cup insert. Consequently, the direction of the force vector was constant relative to the metal back shell cup insert cup and moved relative to the head.

All insert components were artificially aged for 14 days by exposing them to a pure oxygen environment with a temperature level of (70 ± 1)°C and a pressure level of 5.03 bar. Subsequently, all UHMWPE insert components were presoaked in fluid at 37°C for a duration of 14 days.

**Figure 1:**
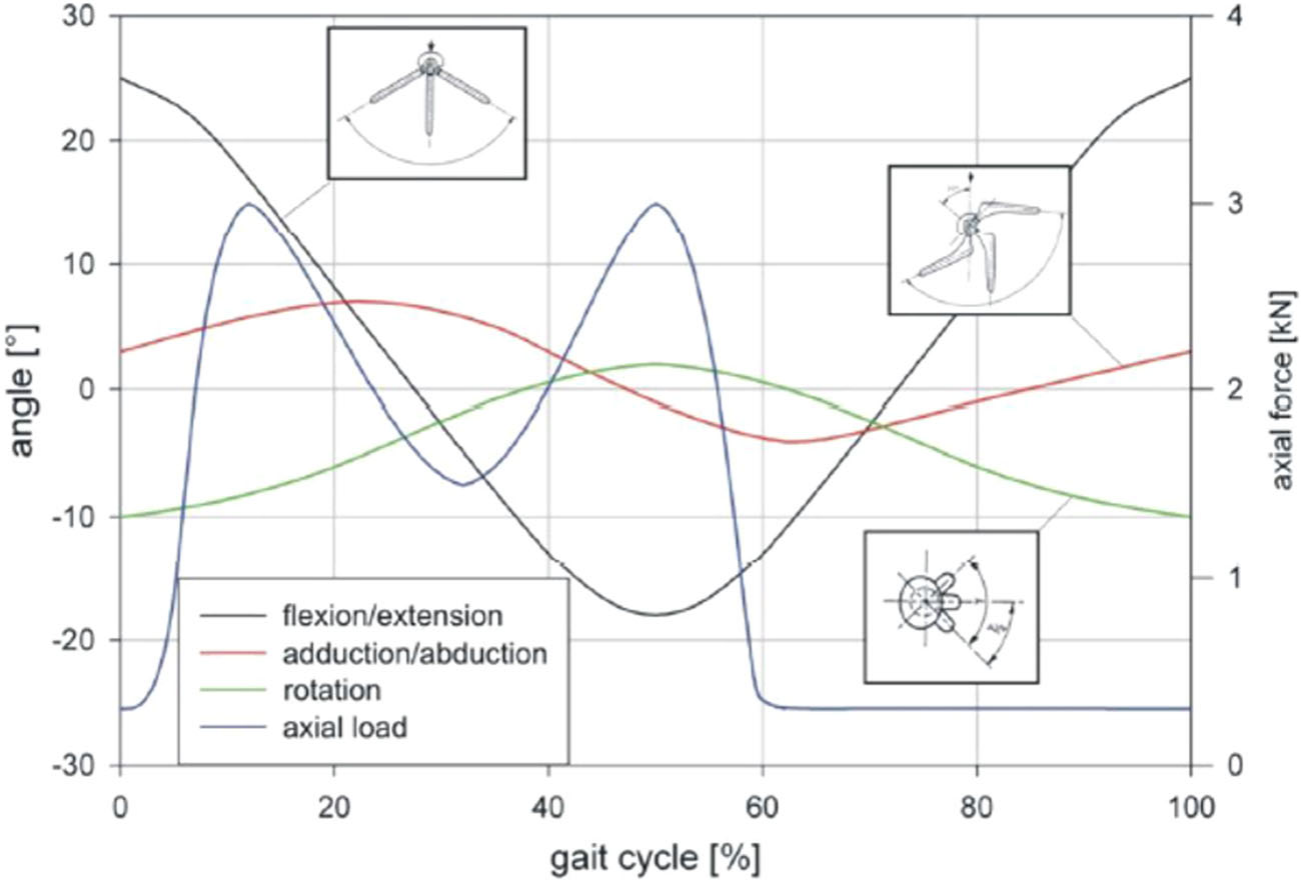
Kinematics and load profile of the ISO 14242‐1 EndoLab® hip simulator.

All three in vivo angular displacements were simulated: Flexion/extension, abduction/adduction, and internal/external rotation. The angular displacements were applied through a gear mechanism driven by an electric motor. The phasing of the individual movements is shown in Figure 1. A maximum load of 3.0 kN was applied (load controlled). The load was actuated by hydraulic cylinders.

The test chamber was filled with approximately 350 mL of test fluid. Testing was stopped every 0.5 million cycles until 5.0 million cycles were reached. Every 0.5 million cycle the test fluid was replaced, the liners were disassembled and weighted to assess mass loss.

To prevent contamination of the cleaned test chambers, the specimen assembly as well as the test fluid injection was conducted within a laminar flow unit (Clean Air). The encapsulated tests chambers were subsequently mounted on the hip simulator.

Calf serum (PAN Biotech GmbH, LOT P141015) diluted with deionized water with a resulting protein content of 30 g/l was used as test fluid. Ethylenediaminetetraacetic (EDTA) was added to the calf serum to bind the calcium phosphate. Amphotericin (250 µg/mL) and gentamicin (10 mg/mL) were added to prevent bacterial and fungal induced degradation. The composition of the test fluid was given in Table 2.

**Table 2:** Composition of the test fluid.

Test fluid composition	
Serum type	Calf
Protein content	30 g/L
Serum	367.6 mL/L
EDTA acid	2.73 g/L
Amphotericin B (250 μg/mL)	10 mL/L
Gentamicin (10 mg/mL)	10 mL/L
Deionized water	632.4 mL/L

Abbreviation: EDTA, ethylenediaminetetraacetic.

### Particle analysis

All tests were performed according to the normative references ISO 17853:2011, ASTM F1877‐16 [3, 22].

Particle analyses were performed on thawed test fluid samples of coupling 1:1 collected after 0.5 and 5.0 million cycles (Mc). According to ISO 14242‐1 [20] the test fluid medium (calf serum) was replaced every 0.5 million cycles. In addition, the analysis was run on a blank sample as an in‐process control.

The test fluids were resuspended by inverting the tubes for about 10 min using a revolver rotator. Next, a sample of 2 mL was taken from each tube. To dissolve biological particles, such as native proteins and partially denatured proteins, a volume of 8 mL 37% HCl was added in accordance with the method described by Scott et al. [30], and resulting solution was continuously and evenly shaken. After digestion of organic matter, 1.0 mL of the solution was diluted with 100 mL methanol. 5 mL of this mixture were filtered through a 0.05 µm polycarbonate filter, using a vacuum pump (Vacuubrand GmbH, ME 4 C NT + 2AK). The filters were sputter coated with silver, and the particles were characterized under a field emission scanning electron microscope (Fe‐SEM) (HITACHI, SU8230). The individual magnification (MAG = 5.000x) and the acceleration voltage (EHT) were recorded for each image. At least 400 particles from five FE‐SEM pictures per filter were analysed. The UHMWPE particles found in the SEM pictures were characterized in size and appearance according to ASTM F1877 [3].

Mean equivalent circle diameter (ECD), form factor (FF), maximum Feret diameter, minimum Feret diameter, area (A), perimeter (p) and aspect ratio (AR) were calculated.

Form factor (FF) was calculated as follows: FF = 4*πA*/*p* ^2^, it varies from 0 to 1 where 1 corresponds to a circle. Feret diameter is the mean value of the distance between pairs of parallel tangents to a projected outline of a particles. The AR is the ratio of the major diameter to the minor diameter: the major diameter is the longest straight line that can be drawn between any two points on the outline, while the minor diameter is the longest line perpendicular to the major diameter.

### Statistical analysis

Descriptive statistics were reported as mean ± standard deviation (SD). Normality of all variables was tested using Shapiro–Wilk test. As regards quantitative wear analysis, a parametric Student's *t*‐test for unpaired data was applied for significant differences between the groups. The qualitative wear data were subjected to the Mann–Whitney test. A *p* value < 0.05 was considered significant. The statistical analyses were conducted using Microsoft Excel (Microsoft) and the XLSTAT resource pack.

## RESULTS

The wears progression showed that a DM group wear lower than a single mobility group as highlighted in Table 3. In fact, the PE CI 69/28 E (DM group) wear was 20.4 mg/Mcy, while in HIPER 28 C (SM group) wear progression was 39.6 mg/Mcy (*p* < 0.01).

**Table 3:** Wear production: Dual mobility versus single mobility.

Cup	Accelerated aging (ASTM F 2003)	Liner	Head	Wear [mg/Mcy]	*p* value
NOVAE E 69 TH	14 days	CI 69/28 E	28/0	20.4	<0.01
HYPE 51 C	14 days	HIPER 28 C	28/0	39.6	

*Note*: Novae E 69 TH: Dual Mobility Cup; Hype 51 C: Single Mobility Cup.

As regards qualitative analysis by scanning electron microscope, a total of 1179 particles were analysed in DM‐PE and 1173 in the SM‐PE. We found out that the wear particles produced in the SM cup has a higher ECD compared to DM cup [(0.24 ± 0.17) μm or (0.22 ± 0.16) μm versus (0.18 ± 0.14) μm or (0.20 ± 0.13) μm]. In addition, the wear particles of the SM cup had a higher area [(0.07 ± 0.11) μm^2^ or (0.06 ± 0.11) μm^2^ versus (0.04 ± 0.08) μm^2^ or (0.05 ± 0.06) μm^2^] and perimeter [(1.18 ± 1.25) μm or (1.03 ± 1.08) μm versus (0.82 ± 0.95) μm or (0.94 ± 0.86) μm].

The maximum and the minimum Feret diameter of wear particles was larger in SM group. On the other hand, the aspect ratio and the form factor resulted higher in the DM group. Wear qualitative analysis data were reported in Table 4.

**Table 4:** Wear qualitative analysis.

Parameter	CI 69/28 E	HIPER 28 C	*p* value
ECD (SD) (μm)	0.18 ± 0.14 0.20 ± 0.13	0.24 ± 0.17 0.22 ± 0.16	n.s.
Area (SD) (μm^2)^	0.04 ± 0.08 0.05 ± 0.06	0.07 ± 0.11 0.06 ± 0.11	n.s.
Perimeter (SD) (μm)	0.82 ± 0.95 0.94 ± 0.86	1.18 ± 1.25 1.03 ± 1.08	n.s.
Form factor (SD)	0.66 ± 0.23 0.64 ± 0.23	0.62 ± 0.24 0.64 ± 0.22	n.s.
Max Feret Ø (SD) (μm)	0.27 ± 0.25 0.31 ± 0.23	0.37 ± 0.32 0.33 ± 0.28	n.s.
Min Feret Ø (SD) (μm)	0.17 ± 0.14 0.19 ± 0.13	0.23 ± 0.17 0.21 ± 0.16	n.s.
Aspect ratio (SD)	1.54 ± 0.36 1.54 ± 0.36	1.52 ± 0.34 1.52 ± 0.34	n.s.

Abbreviations: CI 69/28 E, dual mobility cup liner; ECD, equivalent circle diameter; Feret Ø, Feret diameter; HIPER 28C, single mobility cup liner; SD, standard deviation.

No cases of PErupture were reported. In addition, no ruptures of the acetabular cups and prosthetic stem occurred.

## DISCUSSION

The most important evidence of the present study is that a single‐mobility cup produces a statistically significant greater PE wear than the DM cup. Moreover, the PE wear particles of SM and DM groups are quite similar in terms of shape when analysed with SEM, but PE debris in SM cup resulted to be bigger. Indeed, the wear particles produced in the single mobility prostheses were found to be on average 20% larger in equivalent circle diameter, minimum and maximum Feret diameter and perimeter, even if the statistical significance was not reached. Regarding the shape of debris, as highlighted by the FF and AR, it did not differ between the two groups: ellipsoidal‐shaped. This qualitatively different data of wear between DM and SM cup have never previously been reported.

In the literature, PE debris has been described in different forms (flakes, needles, spears) and most (>90%) of PE particles are less than 1 μm with a spheroidal shape. The size could be related to the specific wear mode: smaller particles are generated when the PE surface is rubbed against bone cement or metals [10, 12].

PE debris is considered to be primarily responsible for triggering a hostile biological response leading to osteolysis and aseptic loosening. Indeed, PE debris can transform appositional bone growth around well‐fixed implants into chronic inflammatory tissue with abundant foreign body giant cells [2].

The bioreactivity of wear particles depends on two main elements: particle characteristics (size, concentration, and composition) and host characteristics [5]

Several studies have been performed concerning the effect of particle shape, size and material on the biological reaction to the same particles. Glant et al. [15] reported a dose‐ and time‐dependent macrophage reaction measured by cytokines, Ti and poly‐methacrylate particles that are phagocytosed. Gelb et al. [14] observed that the larger the total surface area of the particles, the greater the inflammatory reaction.

Regarding the quantitative analysis of wear, our results are in line with what recently published by Haider et al. [17]. In fact, the authors had highlighted how the DM prosthesis combined the benefits of high range of motion with reduced wear and friction.

On the other hand, a study of a comparative analysis based on patient‐specific finite element modelling, reported that a DM cup with a 22.2‐mm‐diameter femoral head produced more volumetric/linear wear when compared with a SM cup with a 22.2 mm diameter femoral head and a lower volumetric wear when compared with SM cup with a 32 mm diameter femoral head. In this study the authors highlighted how the use of a XLPE instead of a conventional UHMWPE drastically reduced the wear, in both the DM and SM prosthesis [32]. The results presented by the authors may differ from ours because of the different methodology used.

Our study is not without limitations. In fact, it is an in vitro study, with a low sample size analysed. Moreover, we evaluated wear in an aged UHMWPE instead of a XLPE and our results may be limited to the acetabular cup and to the femoral head size examined.

## CONCLUSION

This in vitro study suggested that a single‐mobility cup produces twice as much PE wear as a DM cup. In addition, debris is greater in the single‐mobility cup. Further in vitro and in vivo studies are needed to definitively clarify the issue of wear in the dual mobility prosthesis.

## AUTHOR CONTRIBUTIONS

Antonio Pompilio Gigante, Carlo Ciccullo and Luca Farinelli conceived the presented idea. Antonio Pompilio Gigante developed the theory. Marco Senarighi and Luca De Berardinis verified the analytical methods. Marco Senarighi, Luca De Berardinis, Carlo Ciccullo contributed to the design and implementation of the research. Luca Farinelli supervised the project. All authors discussed the results and contributed to the final manuscript.

## CONFLICT OF INTEREST STATEMENT

The authors declare no conflicts of interest.

## ETHICS STATEMENT

All procedures performed in studies involving human participants were in accordance with the ethical standards of the institutional and/or national research committee and with the 1964 Helsinki declaration and its later amendments or comparable ethical standards.

## Data Availability

Available if required.
